# Comparison of the Microtensile Bond Strength of a Polyetherketoneketone (PEKK) Tooth Post Cemented with Various Surface Treatments and Various Resin Cements

**DOI:** 10.3390/ma11060916

**Published:** 2018-05-29

**Authors:** Chan-Hong Song, Jae-Won Choi, Young-Chan Jeon, Chang-Mo Jeong, So-Hyoun Lee, Eun-Sook Kang, Mi-Jung Yun, Jung-Bo Huh

**Affiliations:** 1 Department of Prosthodontics, Dental Research Institute, Institute of Translational Dental Sciences, School of Dentistry, Pusan National University, Yangsan 50612, Korea; chanhong82@gmail.com (C.-H.S.); won9180@hanmail.net (J.-W.C.); jeonycdds@daum.net (Y.-C.J.); cmjeong@pusan.ac.kr (C.-M.J.); romilove7@hanmail.net (S.-H.L.); 2 Department of Prosthodontics, In-Je University Haeundae Paik Hospital, Busan 48108, Korea; prosth-kang@hanmail.net

**Keywords:** polyetherketoneketone (PEKK), post, surface treatment, fiberglass post, microtensile bond strength, resin cement

## Abstract

The aim of this in-vitro research was to evaluate the microtensile bond strength in the newly introduced PEKK tooth post with various surface treatments and resin cements. A fiberglass tooth post was included in order to compare it with PEKK as a possible post material. The microtensile bond strengths of the fiberglass post (FRC Postec Plus) and the PEKK post (Pekkton^®^) were tested using three kinds of self-adhesive resin cements (G-CEM LinkAce, Multilink Speed, and RelyX U200) and one self-etching resin cement (PANAVIA F2.0). The surface treatments of the fiberglass posts were processed according to the manufacturer’s recommendations (F1, application of 37% phosphoric acid etching gel and silanization). For the PEKK post groups, various surface treatments were performed like no surface treatment (P1), sandblasting (P2), silica-coating and silanization (P3), and sandblasting with a composite primer (P4). In the surface treatment, PEKK posts with silica coating and silane treatment (P3) showed a significantly higher microtensile bond strength (mean MPa: 18.09, *p* < 0.05). The highest microtensile bond strength was shown when the PEKK posts were treated with a silica coating and silane treatment and cemented with RelyX U200 (mean MPa: 22.22). The PEKK posts with surface treatments of silica-coating and silanization or sandblasting displayed superior microtensile bond strengths (mean MPa: 18.09 and 16.25, respectively) compared to the conventional fiberglass posts (mean MPa: 14.93, *p* < 0.05).

## 1. Introduction

The dental post-core system is a widely accepted method to maintain the coronal portion of the core after the endodontic treatment of a tooth that has suffered an excessive loss of the coronal structure due to caries or trauma [1]. Ideal posts should have specific physical properties such as the modulus of elasticity, compressive strength and flexural strength similar to those of the structure of a tooth, and they must bond efficiently with dentin and produce esthetic results [2,3]. Posts can produce favorable results if they have a similar modulus of elasticity to the dentin in order to distribute occlusal stress evenly through the pulp cavity to avoid the concentration of excessive stress in the residual dentin [4,5]. Since a metal post has a higher modulus of elasticity than that of dentin, failures such as a root fracture due to the excessive functional stress around the post have been frequently reported [6,7]. Previous studies have reported that materials with a low modulus of elasticity such as fiberglass, allow for the avoidance of root fractures by distributing the occlusal stress [8,9,10,11,12,13,14,15].

However, due to the limitations when placing ready-made fiberglass posts to different morphologies of root canals, the drawbacks of shaping root canals to fit ready-made posts exist. Although fiberglass posts have a lower modulus of elasticity (from 45.7 to 53.8 GPa [16]) than that of alloy posts (110.0 GPa for titanium and 95.0 GPa for gold [17]), the modulus of the elasticity of the fiberglass posts is 3 times higher than that of dentin. Moreover, the major failures in fiberglass posts come from the detachment of the post from the dentin. Herein, a number of surface treatment methods have been investigated with the purpose of enhancing bonding between the post and dentin [18,19,20]. Several studies addressed whether resin cements are appropriate for retaining the post and core. In addition, recent studies have reported that self-adhesive resin cements are favorable to post cementation in narrow root canals [21,22,23].

Recently, PolyEtherKetoneKetones (PEKK), a biocompatible high-performance polymer, has been introduced. PEKK possesses acceptable fracture resistance and its dispersion of stress and shock-absorbing ability presents possibilities for its development as a new restorative material that can replace metals and ceramics [24]. According to research by a PEKK manufacturing company commercializing PEKK as Pekkton^®^ (Cendres+Metaux, Milano, Italia), PEKK (246 MPa) has a similar compression strength to dentin (297 MPa [14]), but a lower modulus of elasticity (5.1 GPa) than dentin [25].

PEKK is considered an attractive material for custom-made dental post-core systems due to its outstanding physical characteristics such as the low modulus of elasticity and the diverse methods of fabrication including milling and pressing. The only study on PEKK as a post-core material was done by Lee et al. through 3D Finite Element Analysis (FEA) [26]. This study reports that PEKK achieves advantages such as the adequate stress distribution and a lower risk of root fracture compared to conventional post-core materials. At the same time, it was reported that PEKK posts present possible debonding between the post and the cement, as well as restorative crown fractures by transferring the stress to the interfacial cement and crown. Therefore, both the post-surface treatment and the cement should be appropriately chosen to achieve a durable bond strength when using PEKK as a post material. Few studies have suggested different surface treatment methods to increase the bond strength of PEKK such as the combination of silica coating and an adhesive primer or the combination of air abrasion and an adhesive primer [27,28]. There has been only one study that performed tensile bond strength tests to evaluate the bond strength between PEKK and resin cements [28]. However, no study has been conducted on the surface treatment and cement bonding of PEKK posts to resist post debonding.

Therefore, the aim of this in vitro research is to evaluate the microtensile bond strength (MTBS) of newly introduced PEKK posts compared to fiberglass posts which are commonly used in clinical situations. The PEKK posts were prepared through various surface treatments and applied with various resin cements. The null hypothesis was that there were no differences in the MTBS between PEKK posts and fiberglass posts with various surface treatments and various resin cements.

## 2. Materials and Methods

In this study, the MTBSs of fiberglass posts (FRC Postec Plus, IvoclarVivadent, Schaan, Liechtenstein) and PEKK (Pekkton^®^, Cendres+Metaux, Milano, Italia) posts were tested using three kinds of self-adhesive resin cements and one self-etching resin cement. The utilized materials are listed in Table 1.

### 2.1. Post Preparation

**Figure 1:**
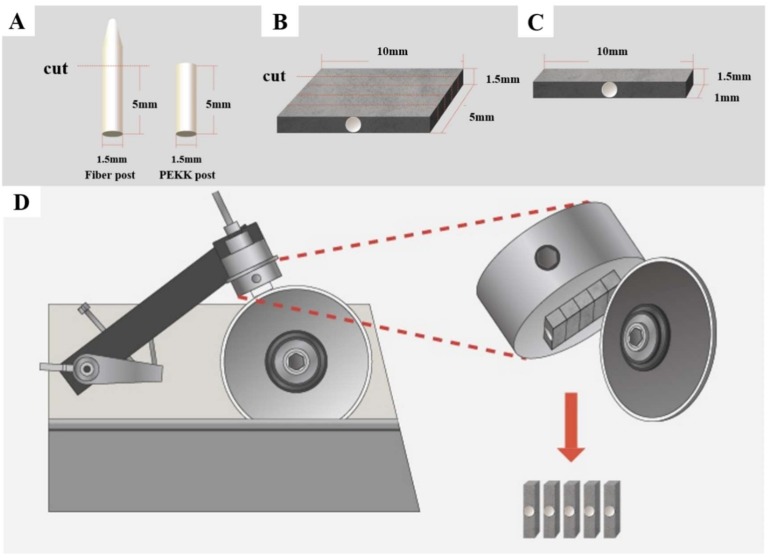
Creating bar type specimens for microtensile bond strength (MTBS) testing; (**A**) the fiberglass and PolyEtherKetoneKetones (PEKK) posts, 1.5 mm in diameter and 5 mm in length, were fabricated; (**B**) the surface-treated post was positioned centrally in the slab type block: 10 mm (L) × 5 mm (W) × 1.5 mm (H); (**C**) the bar type specimen with dimensions of 10 mm (L) × 1.0 mm (W) × 1.5 mm (H) were prepared for MTBS testing; (**D**) using a low speed diamond saw, the slab type block was sectioned into bar type specimens.

The prefabricated fiberglass posts with a diameter of 1.5 mm and 20 mm in length were prepared for this study by removing the tapered portion of the posts and sectioning the cylindrical portion into 5 mm long pieces. The PEKK posts were cast in a cylindrical form that was 5 mm long and 1.5 mm in diameter (Figure 1A). The PEKK posts could be fabricated via either milling or pressing. In this study, they were manufactured through the pressing technique: according to the manufacturer’s instructions, a plastic burnout post (1.5 mm in diameter) was burnt out from the investment material (Bellavest SH, BEGO) at 850 °C and then the PEKKTON ingot (100 g, 35 mm) was pressed at 385 °C. The pressed ingot was then sliced into 5 mm long pieces, essentially resulting in PEKK posts that were 1.5 mm in diameter and 5 mm in length.

### 2.2. Surface Treatments of the Posts

For the fiberglass post group (F1), also known as the control group, the manufacturer’s recommended surface treatment was used: the surface treatment for F1 was achieved by applying 37% phosphoric acid etching gel (Total Etch, Ivoclar Vivadent, Schaan, Liechtenstein) for 60 s, rinsing it with water thoroughly, and drying it. Then the post surfaces were treated with a silane coupling agent (Monobond-S, Ivoclar Vivadent, Schaan, Liechtenstein) for 60 s, carefully dried, and then four cements were used. The surface treatments for PEKK posts were prepared as follows: the P1 group did not undergo any surface treatments and then four cements were used. The P2 group was sandblasted at a pressure of 2–3 bars using 110 µm aluminum oxide (for roughening and enlarging the surface) and then four cements were used. The P3 group was first cleaned with 110 mm aluminum oxide (Rocatec Pre, 3M ESPE, St. Paul, MN, USA), the blasting media. Subsequently, the posts were blasted with silica-modified aluminum oxide (Rocatec Plus, 3M ESPE, St. Paul, MN, USA) at 0.25 MPa for 15 s and they were then cleaned with compressed air for 15 s. The silane coupling agent (ESPE™ Sil, 3M ESPE, St. Paul, MN, USA) was applied next and the posts were dried for 5 min and then four cements were used. The P4 group was sandblasted at a pressure of 2–3 bars using 110 µm aluminum oxide (for roughen, enlarge the surface). The surface was wetted with a composite primer (visio.link, bredent, Senden, Germany) and light-polymerized with an Elipar™ DeepCure-L LED Curing Light for 40 s (3M ESPE, St. Paul, MN, USA) and four cements were applied.

### 2.3. Fabrication of the Specimen

The fabrication method of the specimens used in this experiment is a reference to the fabrication method used in the study by Roperto et al. [29]. All the control and experimental groups were additionally divided into four groups according to the utilized self-adhesive or self-etching resin cement. Cement I was a G-CEM LinkAce (GC, Tokyo, Japan) dual-cure, self-adhesive resin cement. Cement II was a Multilink Speed (Ivoclar Vivadent, Schaan, Liechtenstein) dual-cure self-adhesive resin cement. Cement III was a PANAVIA F2.0 (Kuraray, Osaka, Japan) dual-cure self-etching resin cement. Cement IV was a RelyX U200 (3M ESPE, St. Paul, MN, USA) dual-cure, self-adhesive resin cement. Upon completion of the surface treatment, each post was positioned centrally in the rectangular silicone mold (10 mm (L) × 5 mm (W) × 1.5 mm (H)) and the cavity around the post was completely filled with the resin cement. The resin cements were mixed and applied according to the manufacturer’s instructions. A miscroscopic glass slab was placed on top of the silicone mold to remove the excess resin cement. As all of the four resin cements used in this study were dual-cure, light-curing was performed using a Elipar™ DeepCure-L LED Curing Light (3M ESPE, St. Paul, MN, USA) with an output of 1000–1200 mW/cm^2^ for 40 s on the upper side of the silicone mold through the slide glass. The prepared slab-type block has a post that was positioned at the center with composite resin cement filled on both sides. Each slab-type block was polished using sandpaper to gain an even thickness of 1.5 mm (Figure 1B). Four slab-type blocks were fabricated for each surface treatment group. The slab-type blocks were stored in distilled water for 24 h. Each slab-type block was then attached to an acrylic block with sticky wax and mounted onto the zig of a low-speed diamond saw (Chungdo Inc., Seoul, Korea) (Figure 1C). The block was subsequently sectioned into bar-type specimens with the dimensions of 10 mm (L) × 1.0 mm (W) × 1.5 mm (H) (Figure 1D). A total of 20 bar-type specimens were prepared. The control and experimental groups are summarized in Table 2.

### 2.4. Microtensile Bond Strength (MTBS) Test

Each bar type specimen was glued with a cyanoacrylate adhesive (Zapit; Bisco Inc., Schaumburg, IL, USA) to a MTBS testing machine (Microtensile Tester; BISCO Inc., Schaumburg, IL, USA) and loaded at a crosshead speed of 0.5 mm/min until failure occurred at either side of the bonded interface. The MTBS was obtained by dividing the maximal force applied during the failure test by the bonding surface area. As the bonded interface was curved, the area was calculated using a mathematical formula previously applied by Bouillaguet et al. [30].

### 2.5. Statistical Analysis

The SPSS 23.0 software (IBM Software, Armonk, NY, USA) was used for statistical analysis. The means and standard deviations were calculated and the data were analyzed using the Friedman' test with Tukey’s post hoc test (*p* < 0.05).

### 2.6. Analysis of the Failure Mode and SEM

**Figure 2:**
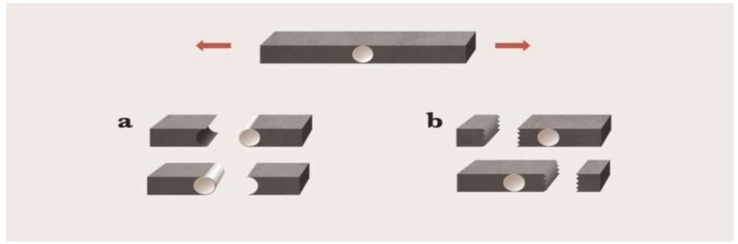
The broken specimens after MTBS testing; (**a**) adhesive failure; (**b**) cohesive failure; (a + b) mixed failure.

After the MTBS test, the bar type specimens were observed using an optical microscope (Olympus BX 51; Olympus, Tokyo, Japan) at 40× magnification for a failure mode analysis (Figure 2). The surface of the PEKK posts with different surface treatments was observed using a field emission scanning electron microscopy (FE-SEM, SUPRA 40 VP, Zeiss) at a magnification of 2000×.

## 3. Results

### 3.1. The Results of the Microtensile Bond Strength Test

The means and standard deviations of the MTBS (MPa) for each of the control and experimental groups were summarized in Table 3. The Tukey test presented significant differences among the control and experimental groups (Table 3). The P3R group showed the highest MTBS value (22.22 ± 3.46 MPa), followed by the P2M group (20.26 ± 2.23 MPa). No significant differences were noticed between the two groups. In addition, the 9 experimental groups that demonstrated the lowest tensile strength—FIR, FIM, P4P, P4G, P2P, P1P, P1M, and P1G—were mostly the fiberglass posts and the groups with PEKK posts without surface treatment, while the groups with PEKK posts with a silica coating and a silane treatment (P3) were not included.

All the PEKK groups with a silica coating and silane treatment (P3) that were cemented with four different resin cements demonstrated superior tensile bond strengths. Among the P2 groups that underwent sandblasting and the P4 groups that underwent sandblasting and the application of a composite primer, the groups cemented with Multilink Speed and RelyX U200, such as P2M, P2R, P4M, and P4R, also showed comparatively high bond strengths. From the fiberglass post groups (the control groups) only the group cemented with PANAVIA F2.0 (F1P) showed a satisfying result. In addition, the PEKK groups without any surface treatment (P1) were not as superior as the 10 experimental groups mentioned above (Table 3).

**Figure 3:**
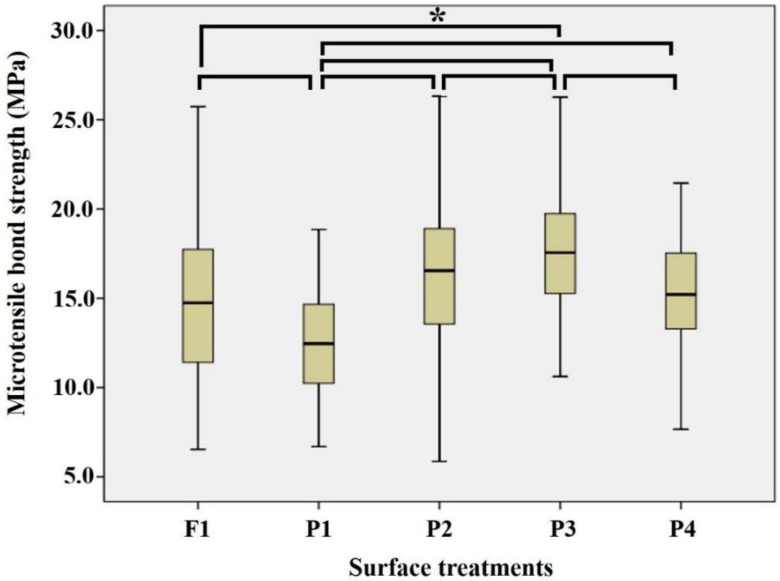
The statistical analysis of the surface treatment regardless of the resin cement used. The mean MTBS value of P3 (surface treatment of the PEKK posts with a silica coating and silanes) showed the highest statistically significant value and compared to the other surface treatment groups. It was followed by P2, R4, F1, and P1. There was no statistically significant difference between P2, P4, and F1. *: statistically significant.

The Friedman test was chosen to examine the experimental groups by surface treatment and Tukey’s test was used to investigate the statistical significance. These tests indicated that the PEKK posts with a silica coating and silane treatment (P3) showed significantly higher tensile strengths (*p* < 0.05). On the other hand, the PEKK posts without any surface treatments (P1) displayed significantly low tensile strengths, that is, values which were lower than the fiberglass post groups (F1, *p* < 0.05, Figure 3).

**Figure 4:**
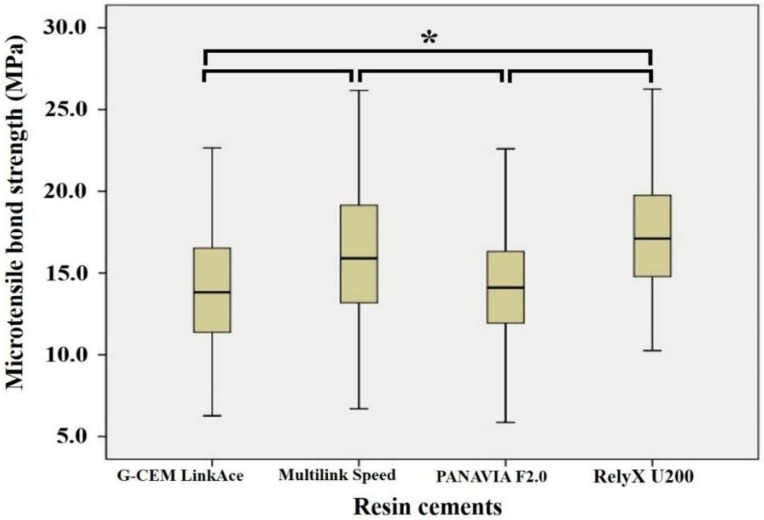
The statistical analysis on resin cement regardless of the surface treatment. The highest mean MPa value was shown with the application of RelyX U200 and there was a statistically significant difference between G-CEM LinkAce and PANAVIA F2.0, but no statistically significant difference shown with Multilink Speed. *: statistically significant.

The experimental groups were also examined by the cement used through the Friedman test and Tukey’s post hoc test. The highest MTBS was derived when cemented with RelyX U200, but this was not significantly different from the values derived from Multilink Speed cement (*p* > 0.05). PANAVIA F2.0, which required an additional application of the primer showed significantly lower MTBS values than the other two types of self-adhesive resin cements (*p* < 0.05); it demonstrated higher MTBS values than G-CEM LinkAce, yet this was not statistically significant *(p* > 0.05, Figure 4).

### 3.2. The Results of the Bonding Failure Mode

The bonding failure mode distribution is shown in Table 4. In this study, the failure modes of the specimens were adhesive failure and mixed failure. Cohesive failure was not observed. For all the groups, a high percentage of adhesive failure was observed, mostly at the interface post-resin cement. The groups P2M, P3M, and P3R showed the highest mixed failure rates (mixed failure mode of 35%), and the groups F1G, P1M, P1G, P1R, and P4G manifested the lowest mixed failure rates (mixed failure mode of 10%). The mixed failure mode rates were greater in the groups with higher MTBS values. The P2, P3, and P4 groups with surface treatment demonstrated more mixed failures than both fiberglass post groups (F1) and the PEKK post groups without any surface treatment (P1).

### 3.3. The Results of the SEM Examination

**Figure 5:**
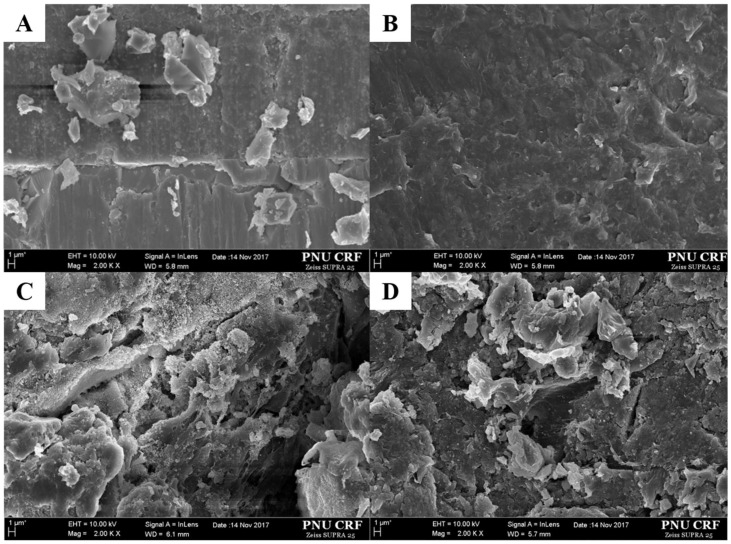
The scanning electron microscopy (SEM) images of different surface treatments at a magnification of 2000×; (**A**) the F1 group: 35% Phosphoric etching; (**B**) the P1 group: no treatment; (**C**) the P2 and P4 groups: alumina particle abrasion; (**D**) the P3 group: silica-coated alumina particle abrasion): in the PEKK post with a surface that was sandblasted with 110 µm aluminum oxide and then blasted with a silica-modified aluminum oxide, irregular particles and a roughness were presented. The mechanical surface pre-treatments tended to form rougher surfaces.

Figure 5 shows the SEM images of the different surface treatments at a magnification of 2000×. The surface of the non-treated PEKK posts manifested a relatively smooth surface with a little roughness (Figure 5B). On the other hand, all the pre-treated PEKK posts, as well as the fiberglass posts, displayed outstanding surface modifications (Figure 5A,C,D). On the surface of the fiberglass post etched with 37% phosphoric acid (F1, Figure 5A), a little irregular particle was observed. On the PEKK post surface that was sandblasted with 110 µm aluminum oxide (P2 and P4, Figure 5C), irregular particles and a roughness were observed. Similarly, on the PEKK post that was surface sandblasted with 110 µm aluminum oxide and then blasted with a silica-modified aluminum oxide (P3, Figure 5D), irregular particles and a roughness were presented. The mechanical surface pre-treatments tended to form rougher surfaces.

## 4. Discussion

The PAEK (polyaryletherketone) family, known for its excellent physical properties and biocompatibility, has been widely used in the medical field [28]. However, the use of PAEK in the dental field is not universal and recent attempts have been made to find new indications. In addition, PEKK, which exhibits the best performance among the polymers of the PAEK family, has been recently introduced [26], but only a few studies have evaluated the bonding strength between PEKK and composites. In the dental clinic, the post and core are frequently used to perform repairs on a tooth lacking a crown, but the detachment of the post is one of the disadvantages that may occur [31,32]. Specifically, the only study on PEKK posts was done through 3D Finite Element Analysis. Therefore, the present study investigated the bonding strength between resin cements and the newly developed PEKK posts fabricated using various surface treatments.

Della Bona et al. reported that tensile bond strength tests are valid when examining interfacial adhesion [33]. The MTBS test was introduced by Sano et al. in 1994 [31]. The conventional macro tensile bond strength test was tested on a large surface area for the adhesion of more than 3 mm^2^ [34]. However, such a broad surface area for adhesion is prone to exaggerate the bond strength. Therefore, Sano et al. suggested an MTBS test using a surface area of 1 mm^2^ for adhesion. According to previous studies, since the tensile force is applied to the minimal surface area for adhesion in the MTBS test, it ultimately derives more significant experimental results than the macro tensile bond strength test by offering a higher possibility to eliminate the defects where stress gets concentrated and dispersing stress evenly [35,36]. Therefore, in this study, the MTBS was measured after the bonding of various surface treated glass fiber posts and PEKK posts to resin cement, mainly due to the adhesion loss between the post and the cement. In particular, a previous study on the PEKK post-core system via 3D Finite Element Analysis remarked that debonding between the PEKK post and cement was feasible and a primary source of failure [26]. Hence, the present study focused on assessing the bonding strength between the post and the cement. Additional bond strength tests, such as the macro-tensile, macro-shear, micro-shear, macro-push out, and micro-push out tests, should be performed for more accurate and significant examination. In order to apply PEKK widely in dentistry, various tests besides the bond strength test should be executed.

According to the study by Fuhrmann et al., the conditioning system and surface texture can significantly affect the tensile bond strength of the PAEK family [28]. The present study also demonstrated distinctive levels of MTBSs by different post surface textures. The P2, P3, and P4 groups with relatively rough surfaces showed higher MTBS values: the interlocking between irregular particles/grooves on the rough PEKK surface and the resin cement is speculated to form mechanical retention. The P3 group, in particular, revealed the most superior tensile bond strength and additional chemical bonding by silica coating and silane treatment which may reinforce the bonding. The covalent bond between the silanol group of the silane coupling agent and the silica-based filler of resin cement and that between the silanol group of the coupling agent and the hydroxyl(–OH) group of silica-coated PEKK post surface seemed to contribute to the additional bond strength. Some studies have also reported that the application of a silane coupling agent enhanced the bonding between the fiberglass posts and the resin composites [37,38]. This experimental result corresponds to those of the research by Fuhrmann et al. in which the greatest tensile bond strength was achieved from the experimental group treated with a silica coating and then with the universal primer (Monobond Plus) [28]. Monobond Plus contains acidic monomers such as sulfidemethacrylates, as well as a silane coupling agent. The acidic monomers are also included in the primers for the self-adhesive resin cement and the self-etching resin cement. Therefore, the data were congruent to that of this study using Monobond-S, which only contains silane, and no significant difference in the bonding strength was presented between any primers.

The previous studies showed that a greater bond strength was accomplished when using one of the commonly used adhesive systems (visio.link) on PEKK [24,39,40]. Moreover, the PEKK manufacturer recommends using visio.link after sandblasting. This is a composite primer with methyl methacrylates (MMA), which is a type of acidic monomer in self-adhesive resin cement. The P4 group where the posts were sandblasted and then treated with visio.link showed lower MTBS values than the P2 group that was merely sandblasted for the following reasons: first, since the three types of self-adhesive cement systems and the one type of self-etching cement system chosen for this study contained functional monomers in the cements while PANAVIA F2.0 incorporated the monomers in the primer, no substantial effect on the bonding strength was presented without applying primers such as visio.link. Secondly, even if primers including visio.link affected the bonding strength, such a chemical treatment hardly enhances the bonding strength when compared to mechanical treatments like sandblasting used in this study and ultimately results in insignificant MTBS differences.

Furthermore, fiberglass posts possessing a comparable modulus of elasticity to dentin were also compared to the PEKK posts as the fiberglass posts are conventionally used in clinical situations with high success rates. Fiberglass posts, conventionally used as a prefabricated post material, face the major problem of post-cement debonding [32]. Herein, a number of studies have been conducted on the tensile bond strength between post-core materials and resin cement. By comparing the tensile bond strength of PEKK to the resin cement measured in this study to that of previous studies, the applicability of PEKK posts may be predicted. According to the study by Ropertoet al, which was designed similar to this study, the highest tensile bond strength of fiberglass posts to resin cement was 13.90 MPa [29]. In the present study, the fiberglass posts (the control group) showed tensile bond strengths in the range of 13.74–16.78 MPa. Furthermore, the PEKK posts of the P1 group displayed MTBS values of 11.00–14.70 MPa, which were lower than the fiberglass posts. In the P2, P3, and P4 groups with a surface treatment, the PEKK posts exhibited a wide range of MTBS values of 13.15–22.22 MPa. The lower measurements (13.15–16.28 MPa) were mostly observed from the posts cemented with G-CEM LinkAce or PANAVIA F2.0 and the higher measurements (15.68–22.22 MPa) were detected from the specimens cemented with Multilink Speed or RelyX U200. Such observations may indicate that the mechanical and chemical surface treatments of the PEKK post and the cementation with either Multilink Speed or RelyX U200 result in a greater bonding strength than that of fiberglass posts.

Compared to the study by Fuhrmann et al. [28], a relatively high MTBS was observed in the present study, which appears to be the consequence of an experimental design constituted without considering artificial aging. Therefore, further studies should establish an experimental design in consideration of artificial aging in order to investigate the bonding durability. In addition, an experimental error may occur due to a premature failure of the specimen and a microcrack on the bonding surface during the manufacturing process of small-sized specimens for the MTBS test.

In addition, this study did not use actual teeth because it was an experiment to recognize the bonding strength with PEKK and resin cement. However, future studies require additional experimental studies using tooth substrates or surrogate models to evaluate the clinical applicability of the PEKK post more realistically.

## 5. Conclusions

Within the limitation of this study, the PEKK posts with a surface treatment displayed superior MTBS values compared to the conventional fiberglass posts. Additionally, when the PEKK posts were blasted with silica-modified aluminum oxide and coated with a silane coupling agent, the bonding strength to the resin cement was enhanced the most effectively. The PEKK posts with sandblasting and those with sandblasting and an adhesive primer application also accomplished satisfying results. Moreover, the proper selection of resin cement can play a critical factor in enhancing the bonding strength of the PEKK post. However, further studies are required to apply the experimental data of this study in a clinical situation and the data may be useful for additional clinical studies in the future.

## Figures and Tables

**Table 1:** The list of materials and their characteristics.

Post Materials (Batch Number)			Main Composition	Manufacturers
Fiberglass post		FRC Postec Plus (U24991)	Glass-fiber-reinforced composite polymer matrix: aromatic and aliphatic dimethacrylates, ytterbium trifluoride	Ivoclar Vivadent, Schaan, Liechtenstein
PEKK post		PEKKTON (0000304681)	PolyEtherKetoneKetones, Titanium dioxide pigments	Cendres+Metaux, Milano, Italia
**Adhesive Materials (Batch Number)**			**Main composition**	**Manufacturers**
Resin cement		G-CEM LinkAce (1608241)	Paste A: Fluoroalumino silicate glass, Initiator, Urethane dimethacrylate (UDMA), Dimethacrylate, Pigment, Silicon dioxide, Inhibitor Paste B: Silicon dioxide, UDMA, Dimethacrylate, Initiator, Inhibitor	GC, Tokyo, Japan
		Multilink Speed (W01675)	Monomer matrix: Dimethacrylates, acidic monomers Inorganic fillers: barium glass, ytterbium trifluoride, co-polymer, highly dispersed silicon dioxide. Catalysts, Stabilizers, Colour pigments	Ivoclar Vivadent, Schaan, Liechtenstein
		PANAVIA F2.0 (000003)	Paste A: 10-Methacryloyloxydecyl dihydrogen phosphate(MDP), Hydrophobic aromatic dimethacrylate, Hydrophobic aliphatic dimethacrylate, Hydrophilic aliphatic dimethacrylate, Silanated silica filler, Silanated colloidal silica, dl-Camphorquinone, Catalysts, Initiators Paste B: Hydrophobic aromatic dimethacrylate, Hydrophobic aliphatic dimethacrylate, Hydrophilic aliphatic dimethacrylate, Silanated barium glass filler, Surface treated sodium fluoride, Catalysts, Accelerators, Pigments	Kuraray, Osaka, Japan
		RelyX U200 (652274)	Base paste: Methacrylate monomers containing phosphoric acid groups, Methacrylate monomers, Silanated fillers, Initiator components, Stabilizers, Rheological additives Catalyst paste: Methacrylate monomers, Alkaline(basic) fillers, Silanated fillers, Initiator components, Stabilizers, Pigments, Rheological additive	3M ESPE, St. Paul, MN, USA
**Materials for Surface Treatment** **(Batch Number)**			**Main Composition**	**Manufacturers**
Chemical	Silica coating	Rocatec Plus (158381)	silica-modified aluminum oxide	3M ESPE, St. Paul, MN, USA
	Silane coupling agent	Monobond-S (U17000)	silane methacrylate	Ivoclar Vivadent, Schaan, Liechtenstein
		ESPE™ Sil (524397)	silane methacrylate	3M ESPE, St. Paul, MN, USA
	Composite primer	visio.link (165127)	Methyl methacrylate, 2-propenoic acid reaction products with pentaerythritol	Bredent, Senden, Germany
Mechanical	Sandblasting	HI-Aluminas (011701)	Aluminum oxide particle	3M ESPE, St. Paul, MN, USA
	Acid etching	Total Etch (W83769)	37% phosphoric acid	Ivoclar Vivadent, Schaan, Liechtenstein

**Table 2:** The control and experimental groups. The fiberglass posts, which are the control groups, were named F1M to F1R according to the resin cements applied after the surface treatment according to the manufacturer’s recommendation. The PolyEtherKetoneKetones (PEKK) posts, which are the experimental groups, were named P1M to P4R, abbreviated according to the surface treatments and the resin cements applied on the PEKK posts. For each group, 20 specimens were used for this test.

Post	Surface Treatment		Resin Cement	Group	n
Fiberglass post (Control)	37% Phosphoric acid + Silane	F1	G-CEM LinkAce	F1G	20
			Multilink Speed	F1M	20
			PANAVIA F2.0	F1P	20
			RelyX U200	F1R	20
PEKK post (Experimental)	No treatment	P1	G-CEM LinkAce	P1G	20
			Multilink Speed	P1M	20
			PANAVIA F2.0	P1P	20
			RelyX U200	P1R	20
	Sandblasting only	P2	G-CEM LinkAce	P2G	20
			Multilink Speed	P2M	20
			PANAVIA F2.0	P2P	20
			RelyX U200	P2R	20
	Silica coating + Silane	P3	G-CEM LinkAce	P3G	20
			Multilink Speed	P3M	20
			PANAVIA F2.0	P3P	20
			RelyX U200	P3R	20
	Sandblasting + Composite primer	P4	G-CEM LinkAce	P4G	20
			Multilink Speed	P4M	20
			PANAVIA F2.0	P4P	20
			RelyX U200	P4R	20

**Table 3:** The results of the microtensile bond strengths test (MPa).

Post	Surface Treatment	Resin Cement	Group	Mean	SD
PEKK	Silica coating + Silane (P3)	RelyX U200	P3R	22.22 ^a^	3.46
PEKK	Sandblasting (P2)	Multilink Speed	P2M	20.26 ^a^	2.23
PEKK	Silica coating + Silane (P3)	Multilink Speed	P3M	18.32 ^b^	3.33
PEKK	Sandblasting + Primer (P4)	RelyX U200	P4R	17.93 ^b^	1.88
PEKK	Sandblasting (P2)	RelyX U200	P2R	16.87 ^b,c^	2.83
PEKK	Silica coating + Silane (P3)	G-CEM LinkAce	P3G	16.28 ^c,d^	1.91
Fiberglass	Etching + Silane (F1)	PANAVIA F2.0	F1P	16.78 ^d^	5.98
PEKK	Silica coating + Silane (P3)	PANAVIA F2.0	P3P	15.54 ^d^	1.76
PEKK	Sandblasting + Primer (P4)	Multilink Speed	P4M	15.68 ^d,e^	3.85
PEKK	Sandblasting (P2)	G-CEM LinkAce	P2G	14.73 ^e^	2.51
PEKK	No treatment (P1)	RelyX U200	P1R	14.70 ^e^	1.85
Fiberglass	Etching + Silane (F1)	RelyX U200	F1R	14.95 ^f^	3.44
PEKK	Sandblasting + Primer (P4)	PANAVIA F2.0	P4P	14.28 ^f^	1.13
Fiberglass	Etching + Silane (F1)	Multilink Speed	F1M	14.27 ^f^	2.52
PEKK	Sandblasting + Primer (P4)	G-CEM LinkAce	P4G	13.79 ^f^	3.34
Fiberglass	Etching + Silane (F1)	G-CEM LinkAce	F1G	13.74 ^f^	4.25
PEKK	Sandblasting (P2)	PANAVIA F2.0	P2P	13.15 ^f^	4.68
PEKK	No treatment (P1)	PANAVIA F2.0	P1P	12.13 ^f^	3.43
PEKK	No treatment (P1)	Multilink Speed	P1M	12.01 ^f^	2.57
PEKK	No treatment (P1)	G-CEM LinkAce	P1G	11.00 ^f^	2.2

The means with the same superscript letter are not statistically different (*p* > 0.05). The mean MTBS values for each group are listed in decreasing order, the superscript letters (“^a^ “ to “^f^“) categorized according to their statistical significance. The statistically significant letters are marked on the mean MPa in order from the highest value. That is, “^a^” represents the most statistically significant MTBS value, and “^f^” represents least the statistically significant MTBS value.

**Table 4:** The failure mode after the microtensile bond strengths tests. For all the groups, adhesive failure was the major failure mode and cohesive failure was not observed. The groups P2M, P3M, and P3R showed the highest mixed failure rate and the groups F1G, P1M, P1G, P1R, and P4G manifested the lowest mixed failure rates.

Group	Failure Rate (%)			Group	Failure Rate (%)		
	Adhesive	Cohesive	Mixed		Adhesive	Cohesive	Mixed
F1G	90	0	10	P2P	85	0	15
F1M	80	0	20	P2R	70	0	30
F1P	70	0	30	P3G	75	0	25
F1R	85	0	15	P3M	65	0	35
P1G	90	0	10	P3P	75	0	25
P1M	90	0	10	P3R	65	0	35
P1P	80	0	20	P4G	90	0	10
P1R	90	0	10	P4M	75	0	25
P2G	70	0	30	P4P	85	0	15
P2M	65	0	35	P4R	70	0	30
